# Advanced diagnostic and therapeutic strategies in nanotechnology for lung cancer

**DOI:** 10.3389/fonc.2022.1031000

**Published:** 2022-12-07

**Authors:** Yujuan Duan, Chen Shen, Yinan Zhang, Yao Luo

**Affiliations:** ^1^ Department of Laboratory Medicine, State Key Laboratory of Biotherapy and Cancer Center, West China Hospital, Sichuan University, Chengdu, China; ^2^ School of Chemical Science and Engineering, Tongji University, Shanghai, China; ^3^ Department of Laboratory Medicine, Shanghai Ninth People’s Hospital, Shanghai Jiao Tong University School of Medicine, Shanghai, China

**Keywords:** nanotechnology, lung cancer, early diagnosis, therapy, potential strategy

## Abstract

As a highly invasive thoracic malignancy with increasing prevalence, lung cancer is also the most lethal cancer worldwide due to the failure of effective early detection and the limitations of conventional therapeutic strategies for advanced-stage patients. Over the past few decades, nanotechnology has emerged as an important technique to obtain desired features by modifying and manipulating different objects on a molecular level and gained a lot of attention in many fields of medical applications. Studies have shown that in lung cancer, nanotechnology may be more effective and specific than traditional methods for detecting extracellular cancer biomarkers and cancer cells *in vitro*, as well as imaging cancer *in vivo*; Nanoscale drug delivery systems have developed rapidly to overcome various forms of multi-drug resistance and reduce detrimental side effects to normal tissues by targeting cancerous tissue precisely. There is no doubt that nanotechnology has the potential to enhance healthcare systems by simplifying and improving cancer diagnostics and treatment. Throughout this review, we summarize and highlight recent developments in nanotechnology applications for lung cancer in diagnosis and therapy. Moreover, the prospects and challenges in the translation of nanotechnology-based diagnostic and therapeutic methods into clinical applications are also discussed.

## Introduction

Lung cancer is a heterogeneous disease that is broadly classified into non-small cell lung cancer (NSCLC) and small cell lung cancer (SCLC), with NSCLC accounting for nearly 85% of all cases (1). Currently, lung cancer is the second most commonly diagnosed cancer worldwide (2). Harmful substances like tobacco smoke and environmental gases are the most significant risk factors for lung cancer (3). Typically, signs and symptoms of lung cancer don’t occur until the disease is already at an advanced stage. In the absence of sensitive detection of cancer occurrence at an initial stage and effective treatments for patients at an advanced stage, lung cancer remains the chief cause of cancer-related death globally in both men and women. It is estimated that approximately 350 people will die from lung cancer each day in the United States, which is more than the combined death rate of breast, prostate, and pancreatic cancers, and 2.5 times more than colorectal cancer, the second leading cause of cancer death (4).

The development of nanotechnology and its application in the field of medicine has provided a new strategy with great potential for the early diagnosis and accurate and effective treatment of lung cancer. Nanotechnology includes a variety of complex theories, methods, techniques, and tools developed at the nanoscale, and the application of numerous nanomaterials. In the past few decades, researchers have discovered/created a variety of multifunctional nanomaterials to improve our quality of life. Some of these nanomaterials are further explored for a wide range of biomedical applications, known as “nanomedicine”, to meet increasingly stringent clinical requirements (5–7). Today, rapid progress has been made in the application of nanotechnology in biomedical fields, including bioimaging, bio-detection, and drug delivery (8). Currently, available nanoplatforms in nanomedicine are mainly divided into three categories, namely organic, inorganic, and organic-inorganic hybrid nanosystems. Due to the great changes in structure, composition, morphology, physical and chemical properties, and biological effects among different nanomaterials, the application of nanotechnology in clinical practice, especially in the field of cancer, has opened up a broad space (6). Nanomaterials combined with probes specifically target tumor tissues, creating the possibility of cancer diagnosis and treatment. Some nanoparticles can be used as drug carriers because their small size helps to penetrate interstitial and mucosal barriers. Nanoparticles can alter the pharmacokinetics, biodistribution, and activity of the therapeutic agents they contain. These properties allow researchers to design specific nanotechnology strategies to improve the therapeutic efficacy of anticancer drugs (9, 10). In addition, some innate biological activities of nanomaterials, such as the regulation of innate and regulatory immune cell functions, can also play an unexpected role in tumor treatment (11). In the field of lung cancer, the integration of nanotechnology will further promote the early diagnosis of tumors. At the same time, nanotechnology creates more possibilities to optimize conventional treatment methods and achieve precise and effective treatment of lung cancer.

**Figure 1:**
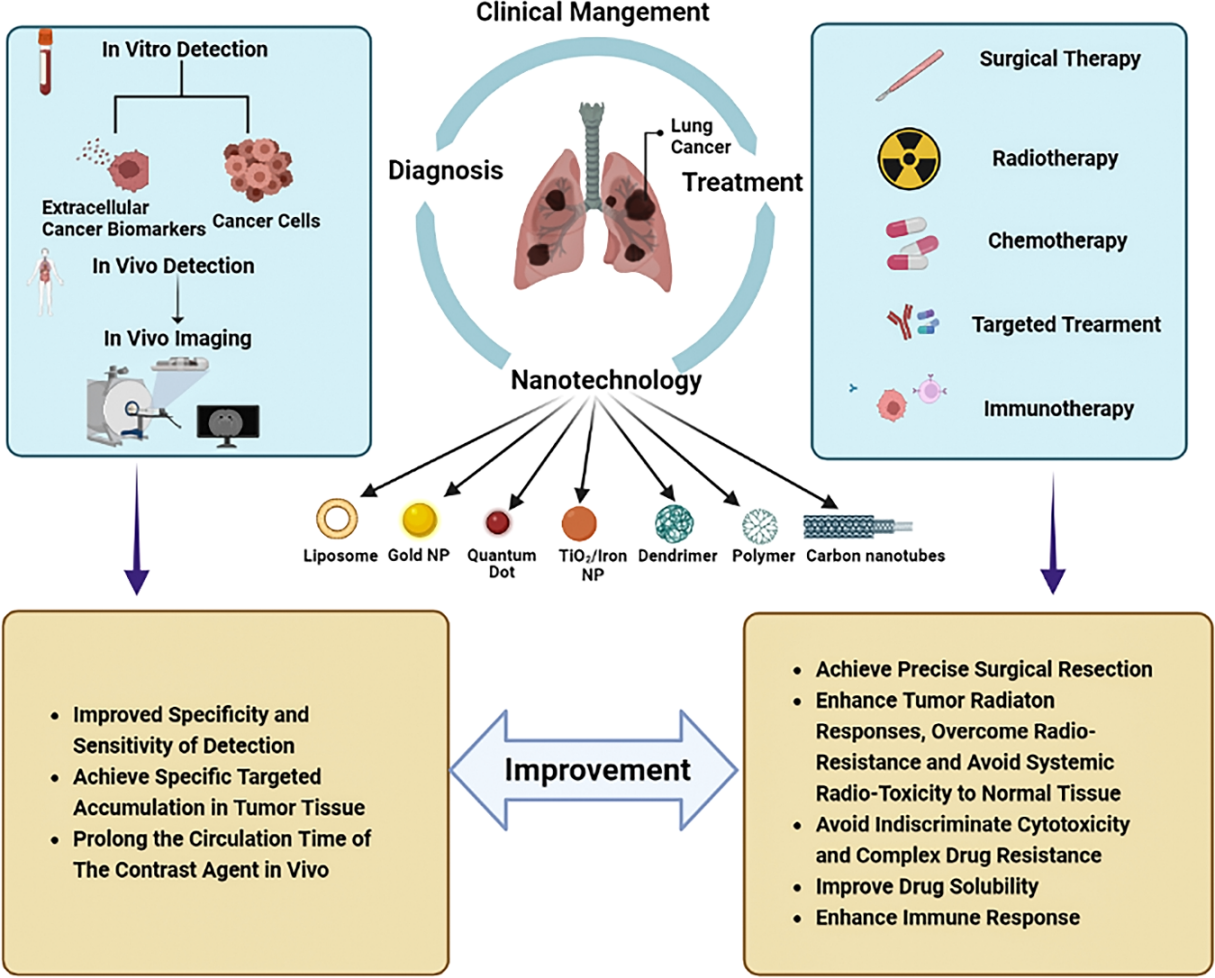
Nanotechnology-based clinical management of lung cancer. The application of nanotechnology in the clinical management of lung cancer includes diagnosis and treatment. For diagnosis, nanotechnology can be used for both *in vitro* detections such as detection of extracellular cancer biomarkers and cancer cells, as well as *in vitro* imaging. In terms of treatment, nanotechnology can be applied in the fields of surgery, radiotherapy, chemotherapy, targeted therapy and immunotherapy. The application of nanotechnology in the management of lung cancer can improve the efficiency of diagnosis and the effectiveness of treatment. For diagnosis, nanotechnology can improve the specificity and sensitivity of detection, achieve specific targeted accumulation in tumor tissues, and prolong the time of contrast media circulation *in vivo*. In the therapeutic field, nanotechnology can help achieve precision surgical resection. Nanotechnology with high specificity can enhance tumor response to radiation, overcome radiation resistance, avoid systemic radiation toxicity to normal tissues, and avoid the occurrence of indiscriminate cytotoxicity and complex drug resistance. In addition, nanotechnology can improve the solubility of drugs and enhance the immune response to tumors.

The existing conventional detection methods for lung cancer, such as chest radiographs (CXRs), computed tomography (CT), magnetic resonance imaging (MRI), sputum cytology, and bronchial biopsy, are heavily dependent on the size and progression of the tumor and may require extra specific medical equipment (12). The most widely used imaging techniques can only detect lung cancer when there is a visible change to the lung fields, by then, thousands of cancer cells may have proliferated and even metastasized. As is crucial to subsequent treatment, the earliest diagnosis of lung cancer has attracted significant interest in research (13). Since the surface of nanoparticles (NPs) can be effectively modified to bind to overexpressed receptors in tumor cells, nanotechnology holds promise as a method with sensitivity, specificity, and multiplexed measurement capabilities for detecting extracellular cancer biomarkers and cancer cells, as well as imaging *in vivo* (**Figure 1**) (14, 15). As demonstrated in studies, early detection with the help of nanotechnology may improve lung cancer survival (16).

Limited options for curative treatment at the time of diagnosis are also the major reason for the poor prognosis of lung cancer. Depending on the stages and subtypes of lung cancer, treatment strategies are different, but surgical therapy, radiotherapy, and chemotherapy remain the top choice for lung cancer. Combined with the molecular type of gene mutations, targeted therapy or immunotherapy gradually become excellent options (17). However, surgery therapy and radiotherapy are both local treatment methods, which only focus on and achieve good curative effects on the primary lesion and local infiltration. Despite the use of chemotherapy, targeted therapy, and immune therapy in the treatment of metastatic lung cancer, systemic toxicity, drug resistance, and immunosuppression remain inevitable (18). Benefiting from the favorable biocompatibility and high specific surface area, nanomaterials can encapsulate chemotherapeutic agents and transport them to lung cancer cells directly to avoid killing normal tissues and reduce side effects (19). Nanoparticles can also overcome drug resistance and/or enhance immune responses to improve the effectiveness of targeted therapy and immunotherapy through their editable and modified characteristics (**Figure 1**) (20–22).

In this review, we summarized and analyzed the potential applications of nanotechnology in improving the early diagnosis and precision treatment of lung cancer, intended to provide an adequate theoretical framework for promoting new diagnosis and treatment options. Moreover, we also discussed current limitations and obstacles that hinder the successful translation of nanomedicines from research to clinical use and tried to analyze how to better chart a course of nanomedicine to enter the clinic and make beneficial differences to lung cancer patients.

## Nanotechnology-based diagnosis of lung cancer

### Nanotechnology promotes *in vitro* diagnosis in clinic

As a biological molecule that can be detected and measured in blood, body fluids, or other tissues, cancer biomarkers including various types of proteins (secreted proteins or cell surface proteins), nucleic acids (circulating tumor DNA, miRNA, etc.), and carbohydrates usually act as a sensitive indicator of cancer occurrence and progression (23, 24). However, the application of biomarkers measurement has been limited by several obstacles, such as the low concentrations of biomarkers in fluids and the heterogeneity in the abundance and timing within patients (25). Due to the large surface-to-volume ratio relative to bulk materials, nanoparticles can be densely covered with proteins, peptides, aptamers, and other small molecules, which is of great significance for the efficient and precise identification and measurement of specific cancer biomarkers (26).

The detection of cancer-specific genetic aberrations in Circulating tumor DNA (ctDNA, approximately 100-200 base-pairs long) released from primary tumors or circulating tumor cells facilitates efficient early diagnosis of lung cancer even before any sign occurs (27). It is reported that plasma is a suitable source of tumor-derived ctDNA for the detection of EGFR alterations in lung tumor patients (28). However, due to the low molecular weights, the enrichment of ctDNA is essential for subsequent tumor progression monitoring. Nguyen et al. present a strategy for ultrasensitive detection of tumor-specific mutations (E542K and E545K) and methylation of ctDNA of the PIK3CA gene based on localized surface plasmon resonance (LSPR) and the coupling plasmon mode of gold nanoparticles (AuNPs) (29). Biocompatible AuNPs, in combination with polymer beads and immunomagnetic beads, have also been developed to facilitate the enrichment of rare ctDNA (30). In addition, nanostructure substrates that offer increased surface areas could facilitate increased interactions with biomolecules, resulting in capturing or isolating ctDNA with high yields and purities (31).

In addition to ctDNA in body fluids, nanoparticles can also be designed to target biomarkers in tumor tissues for accurate *in vitro* diagnosis (32, 33). In recent years, more and more studies have focused on the association between Long non-coding RNAs (lncRNAs) and cancer genesis, progression, and metastasis. Many studies have suggested that some specific lncRNAs can be used as specific markers for tumor diagnosis (34). In lung cancer, the abnormal expression of some lncRNAs can change the tumor immune microenvironment through epigenetic modification and promote carcinogenesis. For example, LncRNA NKILA can regulate the sensitivity of T cells to activation-induced cell death (AICD) by inhibiting NF-κB activity, thereby avoiding immune destruction of cancer cells (35). Specific nanoprobes designed for these lncRNAs can target the tumor microenvironment of lung cancer and achieve early diagnosis of lung cancer through qualitative or quantitative detection (34, 35). Unlike conventional medical methods, nanotechnology can play an unexpected diagnostic role through the unique physicochemical properties of nanoparticles and their interaction with biomarkers. Biomolecules in biological fluids, especially proteins, can form a layer called a “personalized protein corona” on the surface of nanoparticles. Protein corona represents a surface “biotransformation” that inevitably modulates the overall pharmacological and toxicological characteristics of particles in unpredictable ways (36). While “personalized protein crowns” can affect the function of nanoparticles *in vivo*, they are also considered as a biomarker for early disease detection (37). By rationally designing the surface of nanoparticles, the properties and characteristics of protein corona formed in the physiological fluid can be adjusted, to realize the detection of unique protein fingerprints in patients and early diagnosis of tumors (38). Investigators used Gd@C_82_(OH)_22_ nanoparticles, a nanomaterial effective against several types of cancer, as a model nanomaterial to study the native protein fingerprints of personalized protein crowns formed in 10 human lung squamous cell carcinoma patients. Results analysis revealed that a specific biomarker in the corona of lung cancer personalized protein, the complement component C1q, was massively bound to Gd@C_82_(OH)_22_ nanoparticles (38). This new diagnostic strategy reveals the “personalized protein crown” of lung patients, and is also of great guiding significance for the development of precise nano-drugs for lung cancer (38).

### Nanotechnology improves *in vivo* imaging in preclinical cancer model

The specific identification of cancerous tissues in the body has many advantages in the early diagnosis of cancer and subsequent precise treatment. A growing number of studies have shown that the preferential extravasation capacity of 10~150 nm nanoparticles can preferentially accumulate in tumor tissue, as the tight junctions between endothelial cells in new blood vessels in tumors do not form properly, this form of passive nanoparticle entry into the tumor microenvironment is known as the enhanced permeability and retention (EPR) effect. In addition to being specific to tumor tissue, these nanoparticle probes also usually exhibit long circulation times, as well as low toxicity to nearby healthy tissue, allowing them to be used for imaging and diagnosing cancer *in vivo*.

Quantum dots (QDs) are nanometer-size luminescent semiconductor crystals (2~100 nm) characterized by obvious photostability, tunable emission, and high quantum yield, contributing to their wide application in tumor tissue imaging through passive accumulation dependent on the EPR effect. Compared with organic dye molecules, one of the more exclusive properties of QDs is the bright fluorescence and high photochemical endurance, which renders them optimal fluorophores for biomedical imaging (39). Near-infrared (NIR)-emitting QDs, which exhibit high molar excitation coefficients, are particularly well-suited for *in vivo* whole-body imaging techniques because NIR light penetrates the body more deeply than light in the visible spectrum. Researchers have also applied AuNPs to *in vivo* tumor imaging through passive targeting. Lai et al. reported that mercaptoundecanoic acid-coated AuNPs could identify and track cancer cells at the inoculation sites in mice. Furthermore, these particles detected tumor-associated microvasculature in detail. In some cases, chitosan nanoparticles have been used for *in vivo* imaging through the EPR effect (40). Nam et al. reported a tumor-targeting nanoparticle for use as an underlying multimodal imaging probe through optical/MR (MR: magnetic resonance) dual imaging based on self-assembled glycol chitosan (41).

Although nanotechnology has shown great potential for *in vivo* imaging of cancer, current related studies have focused on nanoparticle probe accumulation in tumor tissue for diagnosing cancer in animal models, generally mouse models, and there is still a long way to go for real clinical applications due to safety concerns.

## Nanotechnology-based therapeutics for lung cancer

### Nanotechnology improves the effect of surgical therapy

Surgical resection has always been considered the best curative option for eradication or palliative treatment of lung cancer. As a means of local treatment, surgery mainly focuses on the primary lesion and local infiltration (42). It can be challenging to resect intact lesions in malignant tumors with blurred edges, such as SCLC. Meanwhile, incomplete tumor resection is associated with higher recurrence rates and adverse outcomes (43). Consequently, the development of highly specific and sensitive nanotechnology-based probes for identifying small or incomplete tumors at a cellular level is of great value for removing tumor tissue precisely while conserving normal tissue in surgeries, moreover, the prognosis and survival rates of lung cancer patients can be significantly improved. The ability of nanoparticles to effectively load fluorescein and passively target tumor cells could revolutionize surgical imaging and precise surgical resection (44–46). Additionally, it’s possible that nanorobots could facilitate the complete resection of lesions at the cellular level without surgical incisions (47).

**Figure 2:**
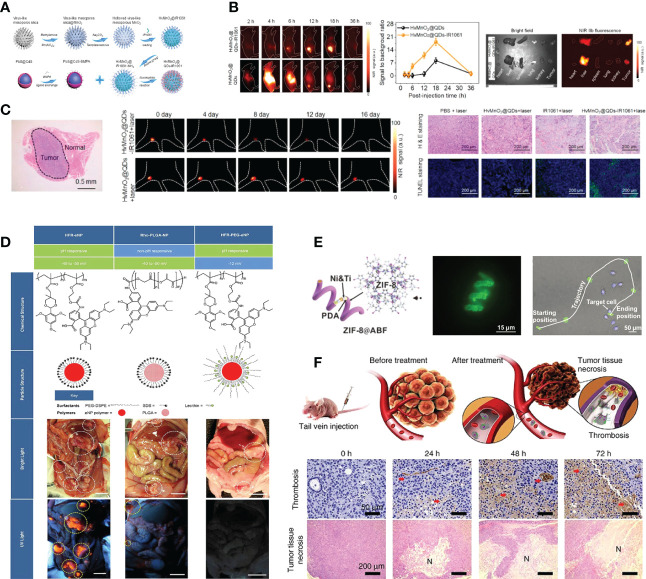
Nanotechnology improves the effect of surgical therapy. **(A)** Model diagram of NIR-IIb quantum dots anchored and IR1061 loaded hollow virus-bionic MnO2 (45). Adapted with permissions from ref (45). Copyright 2022, Elsevier. **(B)** The first two figures show the NIR-IIb fluorescence images of 4T1 breast tumor bearing mice after tail vein injection of HvMnO2@QDs-IR1061 and HvMnO2@QDs for different time points and corresponding SBR. The third figure shows the bright field image (left) and ex vivo NIR-IIb fluorescent image (right) of major organs and tumors after 6 h tail vein injection of HvMnO2@QDs-IR1061 (upper layer) and HvMnO2@QDs (down layer), respectively (45). Adapted with permissions from ref (45). Copyright 2022, Elsevier. **(C)** H & E images of the lymph node metastasis dissection with aid of NIR-IIb fluorescence imaging after tail vein injection of HvMnO2@QDs-IR1061 for 18 h, NIR-IIb fluorescence images of lymph node metastasis after administration of HvMnO2@QDs-IR1061 + laser or HvMnO2@QDs + laser and H & E and TUNNEL images of metastases after different treatments for 4 days (45). Adapted with permissions from ref (45). Copyright 2022, Elsevier. **(D)** Highly fluorescent rhodamine-labeled expansile nanoparticles (HFR-eNPs, yellow circles in UV light images) localize to intraperitoneal tumors (white circles in bright light images) in models of pancreatic, mesothelioma, and ovarian carcinomatoses. All scale bars are 1 cm (46). Adapted with permissions from ref (46). Copyright 2017, American Chemical Society. **(E)** ZIF-8@ABF, Magnified fluorescence image of an RhB@ZIF-8@ABF and Microscopy image showing the movement of an RhB@ZIF-8@ABF structure along a complex trajectory, targeting a single cell (51). Adapted with permissions from ref (51). Copyright 2019, John Wiley and Sons. **(F)** The therapeutic mechanism of nanorobot-Th within tumor vessels. DNA nanorobot-Th was administrated to breast tumor xenografted mice by tail vein injection and targeted tumor-associated vessels to deliver thrombin. The nanorobot-Th binds to the vascular endothelium by recognizing nucleolin and opens to expose the encapsulated thrombin, which induces localized thromboses, tumor infarction and cell necrosis (52). Adapted with permissions from ref (52). Copyright 2018, Nature Publishing Group.

Intraoperative near-infrared (NIR) fluorescence imaging is a novel technique that allows real-time visualization of tumor tissue structures for surgical guidance using a fluorescent contrast agent in conjunction with a dedicated near-infrared camera system (48). In the past few decades, NIR imaging has been used to facilitate resection of solid organ tumors, such as ovarian tumors and liver-metastasized colorectal tumors (49, 50). NIR image-guided surgery has recently been proposed to identify nodules in lung cancers. Okusanya et al. enrolled 18 patients undergoing pulmonary resection for the excision of a single pulmonary nodule. During the preoperative period, all patients received a fine-cut 1mm computed tomography scan, systemic indocyanine green (ICG) 5 mg/kg, followed by an open thoracotomy 24 hours later. Compared to visual inspection or manual palpation, NIR imaging identified 5 additional sub-centimeter nodules on the ipsilateral lung, first providing that NIR imaging is capable of identifying pulmonary nodules from normal tissues and facilitating the tumor resection during thoracic surgery without prior knowledge of their location or existence (44). However, due to the rapid renal clearance, low tumor accumulation and potential toxicity, the biomedical applications of nontargeted NIR dyes remain limited. Recently, with IR1061 incorporated into its cavity and QDs (PbS@CdS) anchored on its surface, Wang et al. reported a tumor microenvironment responsive hollowed virus-bionic MnO2 nanoshell, which can be used in precise NIR-IIb fluorescence imaging guided tumor surgery and efficient NIR-II photothermal therapy (**Figure 2A**). This QD based nanoprobe was capable of adhering on tumor cells to realize tumor tissue accumulating, and the NIR-IIb fluorescence of tumor margin could be specifically delineated after the biodegradation of MnO2 shell for IR1061 release triggered by the weak acid extracellular environment (**Figure 2B**). It was also demonstrated that the eradication of micro-metastases can be possibly achieved *via* NIR-II photothermal effects (**Figure 2C**). Combined with the negligible system toxicity, this versatile NIR-IIb fluorescent imaging platform shows great potential for future clinical translation in lung cancer (45). Similarly, Colby et al. described highly fluorescent rhodamine-labeled expansile nanoparticles (HFR-eNPs) that can be used as visual assistant materials for tumor resection surgery. As a result of the polymer backbone with carboxylate that possesses pH-responsive and negative-potential(-40 to -50 mV) and the covalent incorporation of rhodamine into ∼30 nm eNPs which shows higher fluorescent signal compared to free rhodamine, HFR-eNPs possess the high specificity (99%) and sensitivity (92%) for the detection of sub-centimeter/sub-millimeter tumors under UV light after in transperitoneal injection (**Figure 2D**) (46).

Robotic surgical systems, such as the da Vinci system, allow the surgeon to translate hand movements into smaller, precise movements of tiny instruments located inside patients’ bodies, and are widely used in minimally invasive surgery procedures (53). It has been demonstrated in numerous studies that micro/nanorobots can navigate through complex biological media or narrow capillaries to take images, collect biopsy samples and autonomously release the loaded drug at predetermined locations. Untethered micro/nanorobots represent an attractive alternative to invasive medical robots and are expected to have a significant impact on various aspects of medicine, due to their improved functionality and safety. It is also worth mentioning that many micro/nanorobots are made of biocompatible materials that can degrade or even disappear once their mission has been completed. It is reported that magnetic helical microstructures coated with a kind of zinc-based MOF, zeolitic imidazole framework-8 (ZIF-8), with biocompatibility characteristics and pH-responsive features, are successfully fabricated by Wang et al. The highly integrated multifunctional device can swim along predesigned tracks under the control of weak rotational magnetic fields to achieve single-cell targeting in a cell culture media. The proposed systems can achieve single-cell targeting in a cell culture media and a controlled delivery of cargo payloads (**Figure 2E**) (51). Li et al. constructed an autonomous DNA robot using DNA origami, which delivers thrombin specifically to tumor-associated blood vessels and induces intravascular thrombosis with a DNA aptamer outside that binds nucleolin (a protein specifically expressed on tumor-associated endothelial cells) and the blood coagulation protease thrombin in its cavity, resulting in tumor necrosis and inhibition of tumor growth, proposed a promising strategy for precise drug delivery and preoperative management in cancer therapy (**Figure 2F**) (52).

### Nanotechnology increases the efficacy of radiotherapy

Radiotherapy is another essential local treatment for lung cancer including external beam radiotherapy and internal radioisotope therapy that delivers high-intensity ionizing radiation with high accuracy to the tumor tissue. It is likely that the appropriate dose of radiation is sufficient to kill tumor cells with little harm to surrounding normal tissues, since mitotically active tumor cells are generally slightly more sensitive to ionizing radiations than healthy cells (54). However, the radiation levels may be lower for cancer cells that are further away from the radiation site, and as the development of tumor resistance to the dosed radiation, higher doses are required, ultimately resulting in the death of healthy tissue (55). Additionally, there exists hypoxia in most solid tumors while oxygen is essential to enhance radiation-induced DNA damage, hence the tumor hypoxia-associated radiation resistance is another mechanism that limits the radiotherapy effectiveness (56–58). In conjunction with the advancement in nanotechnology, nanomedicine strategies will hopefully be used to enhance tumor radiation responses and overcome radio resistance (59–62).

**Figure 3:**
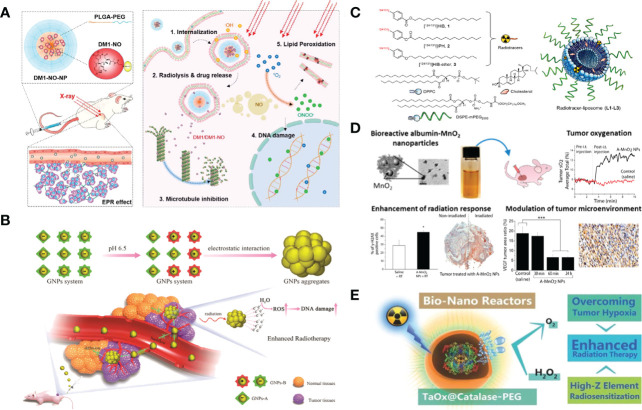
Nanotechnology increases the efficacy of radiotherapy. **(A)** DM1-NO-Encapsulated PLGA-b-PEG Nanoparticles (DM1-NO-NPs) can accumulate in tumors through the EPR effect. In the presence of radiation and/or reduced pH in endosomes/lysosomes, the S−N bond was broken, releasing DM1 and NO. DM1 inhibits microtubule assembly, arresting cells at the more radiosensitive G2/M phase. Meanwhile, NO can react with ROS to form radicals such as peroxynitrites, causing DNA and lipid damage. The combined effects enhance the efficacy of RT (64). Adapted with permissions from ref (64). Copyright 2020, American Chemical Society. **(B)** Diagram depicting the acid-triggered aggregation and composition of GNPs system and schematic illustrations of *in vivo* behavior of GNPs system after intravenous injection for increased tumor retention and enhanced RT (65). Adapted with permissions from ref (65). Copyright 2019, John Wiley and Sons. **(C)** Chemical structures of liposome components and schematic of radiotracer-loaded liposomes (14). Adapted with permissions from ref (14). Copyright 2021, American Chemical Society. **(D)** Multifunctional and colloidally stable bioinorganic nanoparticles composed of polyelectrolyte-albumin complex and MnO_2_ nanoparticles (A-MnO_2_ NPs) and utilized the reactivity of MnO_2_ toward peroxides for regulation of the TME with simultaneous oxygen generation and pH increase. *In vitro* studies showed that these NPs can generate oxygen by reacting with H_2_O_2_ produced by cancer cells under hypoxic conditions. A-MnO_2_ NPs simultaneously increased tumor oxygenation by 45% while increasing tumor pH from pH 6.7 to pH 7.2 by reacting with endogenous H_2_O_2_ produced within the tumor in a murine breast tumor model. Intratumoral treatment with NPs also led to the downregulation of two major regulators in tumor progression and aggressiveness, that is, hypoxia-inducible factor-1 alpha and vascular endothelial growth factor in the tumor. Combination treatment of the tumors with NPs and ionizing radiation significantly inhibited breast tumor growth, increased DNA double strand breaks and cancer cell death as compared to radiation therapy alone (66). Adapted with permissions from ref (66). Copyright 2014, American Chemical Society. **(E)** TaOx@Cat-PEG catalyzes the decomposition of endogenic H_2_O_2_ in the tumor microenvironment to generate oxygen and synergistically enhance therapeutic efficacy of RT (67). Adapted with permissions from ref (67). Copyright 2016, John Wiley and Sons. The symbol ‘*’ means p≤0.05, and ‘***’ means p≤0.001 in statistical reports.

Nanomaterials can act as novel radiosensitizers to enhance the sensitivity of tumor cells to various radioisotopes, thereby promoting the therapeutic effect of lung cancer (63). Gao et al. loaded nitrosylated maytansinoid DM1 (DM1-NO) onto poly (lactide-co-glycolic) - block - poly (ethylene glycol) (PLGA-b-PEG) nanoparticles, allowing DM1 to be targeted delivered to tumors through the enhanced permeability and retention effect. Due to PLGA encapsulation and nitrosylation, the toxicity of DM1 is suppressed. Subsequent radiation elevates the oxidative stress in tumors, leading to the cleavage of the S−N bond and the release of DM1 and nitric oxide (NO). DM1 leads to mitotic arrest and cell enrichment at the more radiosensitive G2/M phase, and NO reacts with reactive oxygen species (ROS) to form radicals such as peroxynitrites that can also effectively contribute to tumor suppression (**Figure 3A**). The radiosensitizing effects were assessed and confirmed in a rodent non-small cell lung carcinoma (NSCLC) tumor model, showing great potential for optimizing radiotherapy of lung cancer (64, 68). In addition, A variety of nanomaterials containing high-Z elements to absorb radiation rays (e.g. X-ray) can act as radio-sensitizers to deposit radiation energy within tumors and promote treatment efficacy. Gold (Z=79) based nanoparticles have been the most widely explored for radio-sensitization, owing to their high inertness, good biocompatibility, ease of chemical modification, and high delivery efficiency (69). Zhang et al. proposed a strategy of acid-triggered aggregation of small-sized gold nanoparticles (GNPs) system within tumor, which results in significant improvement of the radio-sensitization effect in cancer radiotherapy (**Figure 3B**) (65).

Chemically modified nanomaterials can serve as multifunctional nanocarriers to realize *in vivo* targeted delivery of radioisotopes specifically to tumors and avoid systemic radio-toxicity to normal tissues (70). In comparison with free radioisotopes or radiolabeled small molecules, nanoparticles like liposomes are capable of loading a greater dose of radioactivity and a greater variety of radioisotopes inside per particle (71). Moreover, through the EPR effect, or with the additional help of tumor-specific targeting ligands, nanoparticles of suitable size and surface coating would show prolonged blood circulation times to allow enhanced accumulation and retention of radioisotopes in tumor areas (72). Lee et al. described liposomes that are surface-modified with esterase-cleavable radionuclide anchors can greatly reduce radionuclide retention in liver and spleen but remain high radioactivity only in tumors (**Figure 3C**) (14).

It is well recognized that DNA radicals induced by ionizing radiation would react with molecular oxygen to form irreparable DNA damage double strand breaks during radiotherapy (73). Irreparable DNA damages after radiotherapy will be reduced in hypoxic areas of the tumor (74). With the great help of various nanomedicine approaches, the tumor microenvironment could be modulated to overcome hypoxia-associated radiation resistance. Prasad et al. engineered multifunctional and colloidally stable bioinorganic nanoparticles composed of polyelectrolyte-albumin complex and MnO_2_ nanoparticles (A-MnO_2_ NPs), which can effectively increase tumor oxygenation by triggering decomposition of tumor endogenous H_2_O_2_ and significantly inhibit the tumor growth in company with radiotherapy (**Figure 3D**) (66). Song et al. fabricated a novel type of bio-nanoreactor that loaded catalase inside tantalum oxide (TaOx) hollow nanoshells to efficiently supply oxygen through decomposition of endogenic H_2_O_2_. The TaOx nanoshell is capable of not only effectively depositing X-ray irradiation energy within tumor and significantly increasing radiation-induced DNA damage, but also delivering catalase into tumor *via* EPR effect and greatly improving catalytic stability of catalase, which presents an attractive approach for enhancement of radiotherapy (**Figure 3E**) (67).

### Nanotechnology boosts the effect of chemotherapy medications

As a convenient and effective treatment for various cancer, chemotherapy has gained a lot of popularity over the years (75). However, the therapeutic potential of chemotherapy against cancer is seriously dissatisfactory due to the indiscriminate cytotoxicity, short half-life, poor solubility, occurrence of multi-drug resistance, etc. (76). Thanks to its significantly improved therapeutic efficiency and fewer side effects, nanotechnology-based chemotherapy will hopefully overcome these shortcomings and advance the development of lung cancer chemotherapy (77).

**Figure 4:**
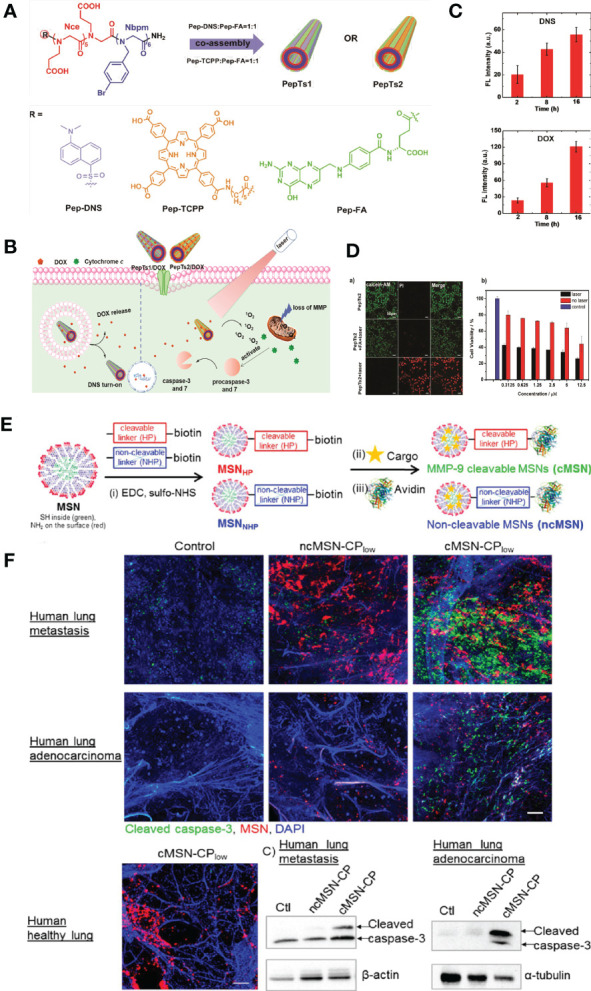
Nanotechnology boosts the effect of chemotherapy medications. **(A)** The structures of ligand-tagged Nbpm6Nce6 peptoid sequences and the assembly of them into 1D tubes. -R represents -Nc6TCPP, -DNS, and -FA, respectively. PepTs1 formed by Pep-FA coassembled with Pep-DNS at a ratio of 1:1. PepTs2 formed by Pep-FA coassembled with Pep-TCPP at a ratio of 1:1 (78). Adapted with permissions from ref (78). Copyright 2019, John Wiley and Sons. **(B)** The endocytosis process of PepTs1 as platform to deliver DOX to H1299 cells, and activation of the apoptosis pathway caused by PepTs2 in H1299 cells (78). Adapted with permissions from ref (78). Copyright 2019, John Wiley and Sons. **(C)** The fluorescence intensity analysis of DOX and DNS for drug release monitoring (78). Adapted with permissions from ref (78). Copyright 2019, John Wiley and Sons. **(D)** Phototoxicity assessment of PepTs2 against H1299 cells. a) live/dead cell images by PI/calcein AM double staining. The laser irradiation power was 660 nm 500 mW cm−2 for 2 min. b) MTT assay of the cytotoxicity of PepTs2/DOX against H1299 cells with/without irradiation at power of 500 mW cm−2 for 5 min (78). Adapted with permissions from ref (78). Copyright 2019, John Wiley and Sons. **(E)** Synthesis scheme of core (green, thiol groups), shell (red, amino groups) functionalized MSNs. (i) EDC amidation of amino groups with carboxy groups of the MMP9 cleavable HP (HP, red) or the MMP9 noncleavable HP-biotin linker (NHP, blue) results in a covalent attachment to the external particle surface (MSNHP, MSNNHP). (ii) After cargo incorporation (cisplatin (CP) or bortezomib combination treatment (CT), yellow star), (iii) the strong binding affinity of biotin to avidin leads to blocking of the mesopores of MSNs with MMP9 cleavable linkers (cMSN) and MMP9 noncleavable linkers (ncMSN) (79). Adapted with permissions from ref (79). Copyright 2015, American Chemical Society. **(F)** Therapeutic effect of MMP9-responsive MSNs in human lungs. Human lung adenocarcinoma and humanhealthy lung 3D-LTC exposed to cMSN-CPlow or ncMSN-CPlow for 72 h. Nonexposed control slices were included in the study.Nuclear staining (DAPI, blue), cleaved caspase-3 (green), and MSNs (red). The scale bar is 50 μm. Images shown are representative for three different sections within the tumor. Western blot analysis of human 3D-LTC exposed to cMSN-CPlow and ncMSN-CPlow for 72 h is also shown in the figure (79). Adapted with permissions from ref (79). Copyright 2015, American Chemical Society.

Luo et al. synthesized two new functional peptoid nanotubes (PepTs1 and PepTs2) by co-assembling ligand-tagged tube-forming peptoids that contain folic acid (FA, an efficient targeting ligand that specifically recognizes FA receptors-overexpressed cells), dansyl (DNS, produce bright green fluorescence after reaction with primary amino groups), and meso-tetrakis(4-carboxy phenyl)porphyrin (TCPP, a photosensitizer used in photodynamic therapy, have the ability to effectively produce cytotoxic ^1^O_2_) (**Figure 4A**). It has been demonstrated that peptoid nanotubes can deliver loaded doxorubicin drugs to targeted tumor cells, PepTs1 have high drug loading efficiency and showed time-dependent, sustainable DOX release in pH 5.5 acidic environment (**Figures 4B, C** **)**. Moreover, activation of the apoptosis pathway can also be caused by PepTs2 in H1299 cells, DOX-loading PepTs2 have high killing efficiency toward H1299 cells due to the combined chemo-photodynamic therapy (**Figures 4B, D** **)** (78).

Van et al. developed avidin-capped mesoporous silica nanoparticles (MSNs) functionalized with linkers that are specifically cleaved by matrix metalloproteinase 9 (MMP9, highly expressed in lung cancer), allowing for controlled release of chemotherapeutics from the MSNs in high MMP9-expressing tumor areas (**Figure 4E**). with the good biocompatibility of MSNs, complex drug resistances and systemic toxicity can be effectively avoided. It has also been demonstrated that the novel system using MMP9-responsive MSNs could be used to effectively control site-selective drug delivery in a combination of two drugs (bortezomib and cisplatin) to achieve the optimal synergistic effect on the therapeutic efficiency of lung cancer (**Figure 4F**) (79).

### Nanotechnology promotes the efficacy of targeted medications

With the rapid development of comprehensive molecular characterization of lung cancer, the understanding of the cellular origins and molecular pathways affected in each subtype has been gradually enhanced (80). These findings provide ‘targets’ for the selection of appropriate adjuvant pharmacological treatment of lung cancer, making ‘precision treatment’ a reality. It has been shown in several studies that the epidermal growth factor receptor (EGFR) mutations-targeted treatment has made encouraging achievements in lung cancer. In comparison with chemotherapy and other traditional therapies, targeted therapy is generally molecular specific, well tolerated, and exhibits little systematic cytotoxicity (81).

By targeting the tyrosine kinase site and binding to the tyrosine kinase receptor domain covalently and irreversibly, epidermal growth factor receptor tyrosine kinase inhibitors (EGFR-TKIs) can block the signaling pathway that activated in various neoplasms, resulting in especially effective treatment for cancer (82). Given the favorable efficacy, EGFR-TKIs have been recommended as standard therapy for advanced lung adenocarcinomas with common EGFR mutations (83). Several EGFR-TKIs have been used as first-line medications in clinical practice, such as gefitinib, afatinib (AFT), erlotinib, dacomitinib, et al. (84). However, due to the capricious oral bioavailability, poor aqueous solubility, initial/acquired drug resistance and off-target side effects, it is difficult to achieve the optimal and complete therapeutic efficacy of EGFR-TKIs in clinical trials. Various nanotechnology-based delivery systems have been gradually developed for allowing efficient delivery of EGFR-TKIs with a better pharmacokinetic profile and tissue-targeting ability (85, 86).

To achieve longer circulation in the blood and an enhanced permeability and retention effect in tumors, Lu et al. encapsulated AFT in liposomes (LPs). Concomitantly, Cetuximab (CTX, an anti-EGFR monoclonal antibody) was also designed to bind to LPs, which also results in the promotion of tumor cell selectivity and therapeutic activity (87). However, owing to insufficient lysosomal escape and uncontrolled drug release, the molecular targeted therapy exhibits insufficient curative effects and often induces various tumor-resistant mutations (88). Zhang et al. reported an active-targeting, enzyme and ROS-dual responsive nanocarrier system (HPGBCA) that comprised of CD44-targeting hyaluronic acid (HA) shells and afatinib (AFT)-loaded, ROS-sensitive poly(l-lysine)-conjugated chlorin e6 (Ce6) derivative nanoparticle cores (PGBCA). Mediated by CD44, AFT and Ce6 can be targeted to tumor cells. with the assistance of ROS generated by Ce6 under NIR irradiation, the intracellular release of AFT can be specifically facilitated. It has been also confirmed that the combination of photodynamic therapy (PDT) and molecular targeted therapy could achieve synergistic therapeutic efficacy in suppressing tumor growth with minimal toxic side effects (89).

Kim et al. synthesized water-soluble erlotinib (NUFS-sErt) by using a nano-particulation platform technology utilizing fat and supercritical fluid (NUFS™) to resolve the low solubility problem that typically prevents the creation of injectable forms of EGFR-TKIs. The effects of NUFS-sErt were similar to those of conventional erlotinib in terms of inhibiting the proliferation of EGFR-mutant lung cancer cells and suppressing EGFR signaling (90). By prolonging circulation time, improving drug solubility, and overcoming drug resistance, Nanotechnology-based targeted therapy has shown great promise for the treatment of advanced lung cancer.

### Nanotechnology enhances immune responses

As an emerging cancer treatment, immunotherapy has shown great potential to target and eradicate cancer cells by orchestrating immune systems, which is likely to result in durable antitumor responses and reduction of tumor metastasis and recurrence (91). It has been demonstrated in previous studies that immune checkpoint inhibitors (ICIs) and adoptive T-cell transfer therapy can improve survival rates in patients with melanoma, non-small-cell lung cancer, and renal cell cancer (92, 93). Ipilimumab plus nivolumab combined treatment was approved for first-line treatment in a subset of patients with advanced non-small cell lung cancer (NSCLC) (94). Nevertheless, there remain many patients who cannot benefit from the immunotherapies for the establishment of an immunosuppressive tumor microenvironment (95). Therefore, strategies to improve overall efficiency and reduce side effects of immunotherapy are much needed. By combining nanotechnology and immunotherapy, we can take advantage of the unique physicochemical properties of nanomaterials and the advantages of efficient tissue-specific drug delivery, enhanced cellular absorption and response to physiological and environmental stimuli to increase the effect of immunotherapy and improve patient outcomes. At present, immunomedicine, as a new field in cancer treatment, has attracted wide interest (10).

**Figure 5:**
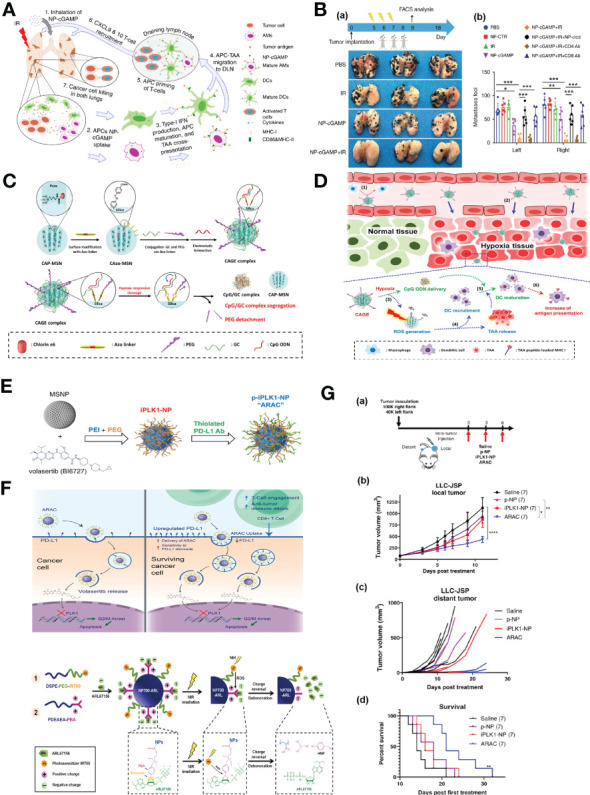
Nanotechnology enhances immune responses. **(A)** The mode of action of the inhalable NP-cGAMP for enhancing antitumor immunity against lung metastases (96). Adapted with permissions from ref (96). Copyright 2019, Springer Nature. **(B)**, (a) The mice (n = 6/group) were sacrificed on day 18 and representative lungs (n = 3) from the treatment groups were displayed. (b) Both lungs were examined under a dissecting microscope and a total of metastatic lung foci on each lung were counted (96). Adapted with permissions from ref (96). Copyright 2019, Springer Nature. **(C)** A synthetic scheme of CAGE complex (97). **(D)** Mode of action of CAGE *in vivo* (97). Adapted with permissions from ref (97). Copyright 2019, American Chemical Society. **(E)** Synthesis scheme of PD-L1 antibody conjugated NP for delivering PLK1 inhibitor volasertib (98). **(F)** Proposed mechanism of action of ARAC nano construct (98). Adapted with permissions from ref (98). Copyright 2022, Springer Nature. **(G)**, (a) 100,000 LLC-JSP cells were injected in right flank and 40,000 cells were injected in left flank of C57BL/6 mice. On day 12 post tumor inoculation, mice (n = 7 per treatment group) received intra-tumoral treatments of saline, p-NP, iPLK1-NP, or ARAC to the right (local) tumor. Each dose consists of 0.5 mg NP (containing 2.5 µg volasertib and/or 20 µg PD-L1 antibody) in 50 µl per dose for 3 doses total. (b) Local (treated) tumor growth. Data presented as mean ± SEM; *P = 0.0104, **P = 0.0017, ****P < 0.0001 (Two-Way repeated measures ANOVA with Tukey’s correction for multiple comparisons). (c) Distant (untreated) tumor growth of individual mice (distant tumors developed in 6/7 saline, 3/7 p-NP, 3/7 iPLK1-NP, and 2/7 ARAC at shown time-points). (d) Kaplan–Meier Survival curve (mice were euthanized when a combined tumor size reached 2000 mm3). **P = 0.0036 for ARAC vs. saline (Log-rank Mantel–Cox test) (98). Adapted with permissions from ref (98). Copyright 2022, Springer Nature. **(H)** Scheme illustration of the self-assembly of NP700-ARL (99). Adapted with permissions from ref (99). Copyright 2022, The American Association for the Advancement of Science.The symbol ‘***’ means p≤0.001 in statistical reports.

The extent of antigen presentation by antigen presenting cells (APCs), which phagocytize tumor-associated antigens (TAA) in the tumor site and migrate to tumor draining lymph nodes (TDLN) for the activation of T cells, is very crucial for successful cancer immunotherapy (CIT). Liu et al. reported an phosphatidylserine (PS) coated inhalable liposome loaded with STING agonist cyclic guanosine monophosphate–adenosine monophosphate (NP-cGAMP) enables targeted delivery of immunostimulants to pulmonary intratumoral APCs to enhance anticancer immunity against lung metastases. PS on the surface of NP-cGAMP can be recognized and engulfed by macrophages and dendritic cells (DCs). After ingestion by APCs, cGAMPs can be cytosolic released in response to low endosomal pH, stimulating STING signaling and type I interferons production in APCs, resulting in the formation of pro-inflammatory tumor microenvironment and recruitment of cytotoxic CD8^+^ T cells in multifocal lung metastases (**Figure 5A**). It has also been observed that intratumoral injection of NP-cGAMPs alone or synergized with irradiation (IR) has shown its aptitude for enhancing antitumor immunity (**Figure 5B**) (96). Im et al. developed a hypoxia-responsive photosensitizer that denoted as chlorin e6 (Ce6)-doped-azobenzene-glycol chitosan (GC)-PEG mesoporous silica nanoparticle (CAGE) for an enhanced CIT assisted by photodynamic therapy (PDT) (**Figure 5C**). CAGE was designed to improve the intracellular uptake of nanocarriers and the delivery of adjuvants to DCs, and PDT was exploited for the generation of immunogenic debris and recruitment of DCs in a tumor site, followed by enhanced antigen presentation, which resulted in incomparable antitumor effect *in vivo* (**Figure 5D**) (97).

Immune checkpoint inhibitors (ICIs) targeting programmed death 1 (PD-1) and PD-ligand 1 (PD-L1) have made a significant breakthrough in the management of lung cancer. However, only a minority of NSCLC patients respond to ICIs, superior immunotherapies are urgently needed. Reda et al. reported a nano-immunotherapy termed ARAC (Antigen Release Agent and Checkpoint inhibitor) for co-delivery of a PLK1 inhibitor (volasertib) and PD-L1 antibody, which is built upon a polymer-coated mesoporous silica nanoparticle (NP) platform that can effectively achieve the therapeutic synergy of the drug combination (**Figure 5E**). The PLK1 inhibition upregulates the expression of PD-L1 in cancer cells, thereby diminishing the function of cytotoxic T cell. Accordingly, the combination of PD-L1 blockade with PLK1 inhibition can significantly reduce tumor burden and prolongs survival of lung tumor-bearing mice (**Figures 5F, G** **)** (98). Mao et al. proposed that the tumor evasion of immune destruction is associated with the production of immunosuppressive adenosine in the tumor microenvironment (TME), thereby, they constructed reactive oxygen species (ROS)-producing nanoparticles that loaded with ARL67156 (ectonucleotidase inhibitor) through electronic interaction and phenylboronic ester. Upon near-infrared irradiation, the nanoparticles-produced ROS induced the release of ATP from cancer cells *in vitro* and triggered the cleavage of phenylboronic ester, resulting in ARL67156 release, ARL67156 prevented conversion of ATP to adenosine and enhanced anticancer immunity (**Figure 5H**), Nanoparticle-based ROS-responsive drug delivery system reprogramed the immunogenic landscape in tumors, eliciting tumor-specific T cell responses and tumor regression, conferring long-term survival in mouse models (99).

## Conclusion and future perspective

Over the past few decades, the development and advances of nanotechnology have received considerable attention and interest in the field of clinical management of lung cancer (**Table 1**). The application of several nanoparticles could significantly improve the sensitivity and selectivity of cancer diagnosis, both in the detection of biomarkers *in vitro* and in the enhancement of imaging *in vivo*. By providing better encapsulation and bioavailability, controlled release, and lower toxicity, nanotechnology-based therapeutics are able to overcome the inherent limitations of traditional lung cancer treatment, including drug resistance, systemic toxicity, rapid clearance rate, etc (100). The multimodal treatment method of nanotechnology combined with traditional methods are becoming the trend.

**Table 1:** Overview of nanotechnology for the diagnosis and treatment of lung cancer.

Name	Composition	Application	Ref.
LSPR-AuNPs	PNA, AuNPs	Detect the hot-spot mutation and epigenetic changes on the ctDNA	(29)
Gd@C_82_(OH)_22_	Gd@C_82_(OH)_22_	Detect the “personalized protein crown” of lung patients	(38)
NIR-emitting QDs	QDs	*In vivo* imaging	(39)
NIR image-guided surgery	IR1061, PbS@CdS, MnO2 shell	Facilitate resection of solid organ tumors	(45)
HFR-eNPs	Carboxylate, rhodamine, eNPs	Act as visual assistant materials for tumor resection surgery he high specificity and sensitivity	(46)
ZIF-8	zinc-based MOF (ZIF-8)	Achieve single-cell targeting in and a controlled delivery of cargo payloads	(51)
DNA origami robot	DNA origami	Deliver thrombin specifically to tumor-associated blood vessels and induce intravascular thrombosis	(52)
DM1-NO PLGA	DM1-NO, PLGA-b-PEG	Work synergistically to enhance radiotherapy outcomes	(64)
Acid‐triggered aggregation GNPs	GNPs	Act as radio-sensitization	(65)
Esterase-cleavable radionuclide-modified liposomes	Liposomes, radionuclide	Enhance accumulation and retention of radioisotopes in tumor areas	(14)
A-MnO2 NPs	Polyelectrolyte-albumin complex, MnO2 NPs	Increase tumor oxygenation, enhance radiotherapy	(66)
Catalase-TaOx	TaOx nanoshell,	Enhance radiotherapy	(67)
PepTs1/PepTs2	FA, DNS, TCPP	Have high drug loading efficiency and improve the therapeutic efficiency of chemotherapy	(78)
avidin-MSNs	MSNs, avidin	Allow for controlled release of chemotherapeutics from the MSNs in high MMP9-expressing tumor areas	(79)
AFT-CTX-LP	AFT, CTX, liposomes	Promote the tumor cell selectivity and therapeutic activity of the targeted therapy	(87)
HPGBCA	HA, AFT, PGBCA	Combine photodynamic therapy (PDT) and molecular targeted therapy, to achieve synergistic therapeutic efficacy with minimal toxic side effects	(89)
PS coated NP-cGAMP	PS, cGAMP, NP	enables targeted delivery of immunostimulants to pulmonary intratumoral APCs	(96)
CAGE	Mesoporous silica nanocarrier	Improve the intracellular uptake of nanocarriers and the delivery of adjuvants to DCs	(97)
ARAC	Polymer, mesoporous silica NP	Co-delivery of a PLK1 inhibitor (volasertib) and PD-L1 antibody	(98)
NP700-ARL	ARL67156, phenylboronic ester, lipid polymer	Reprogram the immunogenic landscape in tumors, elicit tumor-specific T cell responses and tumor regression	(99)

LSPR, localized surface plasmon resonance; AuNPs, gold nanoparticles; PNA, Peptide nucleic acids; Gd@C_82_(OH)_22_, Gadolinium metallofullerenol; QDs, Quantum dots; NIR, near-infrared; HFR-eNPs, highly fluorescent rhodamine-labeled expansile nanoparticles; ZIF-8, zeolitic imidazole framework-8, a zinc-based MOF; DM1-NO, nitrosylated maytansinoid DM1; PLGA-b-PEG, poly(lactide-co-glycolic)-block-poly(ethylene glycol); GNPs, gold nanoparticles; A-MnO2 NPs, nanoparticles composed of polyelectrolyte-albumin complex and MnO2 nanoparticles; TaOx, tantalum oxide; FA, folic acid; DNS, dansyl; TCPP, Meso-tetrakis (4-carboxy phenyl) porphyrin; MSNs, mesoporous silica nanoparticles; AFT, afatinib; CTX, Cetuximab; HA, CD44-targeting hyaluronic acid shells; PGBCA, ROS-sensitive poly (l-lysine) -conjugated chlorin e6 (Ce6) derivative nanoparticle cores; PS, phosphatidylserine; cGAMP, STING agonist cyclic guanosine monophosphate–adenosine monophosphate; CAGE, chlorin e6 (Ce6)-doped-azobenzene-glycol chitosan (GC)-PEG mesoporous silica nanoparticle; ARAC, Antigen Release Agent and Checkpoint inhibitor, a nano-immunotherapy for co-delivery of a PLK1 inhibitor (volasertib) and PD-L1 antibody, which is built upon a polymer-coated mesoporous silica nanoparticle (NP) platform; ARL67156, ectonucleotidase inhibitor.

A growing number of nanomedicines are gaining regulatory approval and have great potential for clinical translation. However, the application of nanotechnology in clinical practice still faces some challenges and requires further in-depth research. First, we currently know very little about the pharmacokinetics (*in vivo* circulation, distribution, metabolism, excretion, etc.) of nanomaterials. Although some studies have shown that some nanomaterials can maintain structural integrity for a long time *in vivo* (101). However, it remains unclear how the physical and chemical properties of these nanomaterials affect the pharmacokinetic bioavailability. This makes it difficult for clinicians to grasp the dosage and timing of nanomedicine use, while bringing unpredictable drug side effects. Secondly, although many medical nanomaterials show strong specificity in research, considering the extremely small size of nanomaterials, it is easy to be taken up by non-target cells through active transport. This may lead to unnecessary effects or damage of nanomedicine on normal tissue cells and reduce the efficacy of nanomedicine. Therefore, designing methods for selective uptake of nanomaterials by specific organs and diseased cells while minimizing nonselective uptake by normal organs and cells is a challenge for nanotechnology researchers (102). However, given that nanomaterials are fully programmable and precisely controllable, this challenge will hopefully be overcome. In fact, both the unknown pharmacokinetics and the nonspecific tissue cell binding will bring about concerns about the biosafety of nanomedicine. Ensuring safety is the most important prerequisite for the application of nanotechnology in clinical practice. Although some substances such as DNA are biodegradable and biocompatible, when they are modified into nanostructures, changes in their physicochemical properties may have unknown effects. In addition, nano-metal particles are also very commonly used nanomaterials. Researchers should pay attention to the cytotoxicity and metabolism of these metals *in vivo*. The potential immune stimulatory characteristics and adverse immune reactions caused by nanomaterials should also be explored in depth in the process of research. Therefore, any nanomaterials should undergo systematic experiments, verify their safety and undergo strict review by relevant departments before being used in clinical practice. In general, mouse xenograft cancer models are widely used to perform *in vivo* mimics of nanodrugs, but it is difficult to accurately simulate the immune landscape of an orthotopic tumor in a subcutaneous model of the tumor. In addition, the heterogeneity of human diseases affects the biodistribution of nanomedicine, which is also difficult to simulate and replicate in animal models. Therefore, the safety of nanomedicine is still the biggest challenge in the process of clinical transformation of nanotechnology. Finally, the cost of producing some nanomedicines is a concern. For practical biomedical applications, we need to be able to produce functional nanomaterials with high purity, which often means an increase in cost (8). At present, there are not many researches on low-cost nanomedicine, but this is a problem that must be faced in the process of clinical translation of nanotechnology. How to optimize the synthesis method of nanomedicine, find cheaper raw materials, and achieve large-scale low-cost production are worthy of further research.

## Author contributions

YL and YZ designed this study. YD drafted the manuscript. CS performed drawing and organization of figures. YL, YZ, YD and CS revised the manuscript. All authors contributed to the article and approved the submitted version.
